# Magnetic Nanovectors for the Development of DNA Blood-Stage Malaria Vaccines

**DOI:** 10.3390/nano7020030

**Published:** 2017-02-10

**Authors:** Fatin M. Nawwab Al-Deen, Sue D. Xiang, Charles Ma, Kirsty Wilson, Ross L. Coppel, Cordelia Selomulya, Magdalena Plebanski

**Affiliations:** 1 Department of Chemical Engineering, Monash University, 18 Alliance Lane, Clayton, VIC 3800, Australia; fatin.mn70@gmail.com; 2 Department of Immunology and Pathology, Central Clinical School, Faculty of Medicine, Nursing and Health Sciences, Monash University, 89 Commercial Road, Melbourne, VIC 3004, Australia; kirsty.wilson@monash.edu; 3 Department of Microbiology, Monash University, Wellington Road, Clayton, VIC 3800, Australia; charles.ma@monash.edu (C.M.); ross.coppel@monash.edu (R.L.C.)

**Keywords:** hyaluronic acid, MSP1_19_, superparamagnetic iron oxide nanoparticles (SPIONs), magnetic gene vector, malaria DNA vaccine, antibody, immune response

## Abstract

DNA vaccines offer cost, flexibility, and stability advantages, but administered alone have limited immunogenicity. Previously, we identified optimal configurations of magnetic vectors comprising superparamagnetic iron oxide nanoparticles (SPIONs), polyethylenimine (PEI), and hyaluronic acid (HA) to deliver malaria DNA encoding *Plasmodium yoelii* (Py) merozoite surface protein MSP1_19_ (SPIONs/PEI/DNA + HA gene complex) to dendritic cells and transfect them with high efficiency in vitro. Herein, we evaluate their immunogenicity in vivo by administering these potential vaccine complexes into BALB/c mice. The complexes induced antibodies against PyMSP1_19_, with higher responses induced intraperitoneally than intramuscularly, and antibody levels further enhanced by applying an external magnetic field. The predominant IgG subclasses induced were IgG2a followed by IgG1 and IgG2b. The complexes further elicited high levels of interferon gamma (IFN-γ), and moderate levels of interleukin (IL)-4 and IL-17 antigen-specific splenocytes, indicating induction of T helper 1 (Th1), Th2, and Th17 cell mediated immunity. The ability of such DNA/nanoparticle complexes to induce cytophilic antibodies together with broad spectrum cellular immunity may benefit malaria vaccines.

## 1. Introduction

DNA vaccines, often described as “third generation” vaccines, offer a new approach for the prevention and therapy of several diseases of both bacterial and viral origin [1,2,3], and have been widely used in laboratory animals and non-human primates to induce protective antibody and cellular immune responses [4]. Compared to conventional immunisation strategies, which commonly use live-attenuated pathogens, proteins, or synthetic peptides, DNA-based immunisation exhibits several important advantages and incorporates many of the most attractive features of each approach, such as: (1) eliciting humoral and/or cellular immune responses directly without the need for live vectors or complex biochemical production techniques [5]; (2) highly specific, and the expressed target antigen is subjected to the same glycosylation and post-translational modifications as natural intracellular infections [6]; (3) as a platform, it is capable of incorporating multiple variants of an antigen into a single array within a plasmid vaccine [7,8,9]; and (4) relative ease and low cost of production and transportation makes them highly suited for production and distribution in the developing world than other systems. Although DNA vaccines have been shown to be effective in some animals [10], the human clinical potency of DNA vaccines has been disappointing, overall, with no human vaccines licensed to date [11]. This is mostly due to the low levels of antigen-specific immune responses induced by naked DNA immunisations. It is expected this problem can be overcome by using delivery systems and devices/carriers which can deliver the DNA effectively across the different biological barriers. This would then facilitate the eventual localisation of DNA into intracellular compartments that enhance gene expression in the antigen-presenting cells (APCs) most capable of causing downstream potent antigen-specific immune cell activation [11]. 

The development of nanocarrier-based DNA vaccine formulations has become one of the most prominent areas of vaccine research [12]. Different studies have verified that nonviral carrier systems in the nanometer-size range can be successfully used as transfection agents to deliver nucleic acids to APCs for both in vitro and in vivo applications [12]. Magnetic particle-assisted gene delivery, also known as magnetic transfection or magnetofection, has been proven to increase both the efficiency and the speed of gene delivery in different tissues [13]. We have synthesised superparamagnetic nanoparticles (SPIONs, 80 ± 5 nm), which deliver malaria DNA via magnetofection, and efficiently transfected eukaryotic cells in vitro [14].

Malaria is one of the most severe public health problems worldwide. Although the number of malaria cases and deaths have been falling since 2000, due to worldwide efforts (World Health Organization (WHO) report) [15], in 2015 there were still an estimated 214 million clinical cases (decline of 18%), and 438,000 deaths (decline of 48%), with 88% of deaths in Africa [15]. Despite this tremendous progress, more needs to be done to further reduce the malaria burden. However, in spite of many decades of intense research and development efforts, there are currently no commercially available malaria vaccines, though, more than 20 vaccine constructs are being evaluated in preclinical and clinical studies. Of particular interest, merozoite surface protein 1 (MSP1), in particular, the C-terminal 19 kDa region (MSP1_19_), is regarded as a promising and leading vaccine candidate [16]. MSP1 is an abundant and essential protein present in approximately all species of malaria parasites that is carried into red blood cells during merozoite invasion [17]. Several in vitro and in vivo studies have shown that MSP1_19_ is the target of protective immune responses against asexual blood stages of malaria parasites [18,19], and antibodies against MSP1_19_ are thought to act through the direct inhibition of merozoite invasion into red blood cells [20]. Our own studies on MSP1_19_ as a vaccine target have shown that nanosized magnetic vectors (SPIONs/ polyethylenimine (PEI)/DNA gene complexes, 100–300 nm) containing the gene encoding MSP1_19_ (VR1020-PyMSP1_19_ gene complexes) were immunogenic, and capable of evoking the desired antibody responses [21]. To further improve the stability of the DNA formulation construct, as well as to enhance the intrinsic transfection activity, we produced new constructs incorporating hyaluronic acid (HA) (SPIONs/PEI/DNA + HA gene complexes). HA has been shown to interact with multiple receptors on key antigen-presenting cells such as dendritic cells (DCs) and macrophages, including Toll-like receptors (TLRs) such as TLR2, TLR4, and tumour necrosis factor-induced protein-6 (TNFIP6) [22,23]. Moreover, HA is a ligand for CD44 expressed at high levels on haematopoietic cells, including immune cells, which can be used to additionally promote cellular uptake via this receptor [24,25]. Such new constructs show enhanced stability [26], and are efficiently taken up by and transfect DCs [27].

As well as identifying a useful formulation which is capable of transfecting DCs, DNA vaccines may differ in efficacy across different routes of administration. Furthermore, their ability to be captured by APCs may potentially be enhanced under magnetic fields by stabilising local SPION concentrations to the site of injection, creating a “depot effect”. Based on the above, we have designed a synthetic gene delivery system with multiple prosperities for efficient gene transfection, by using magnetic nanoparticles coated with the polymer ligand HA, accompanied by an external magnetic field applied during gene transfection. These formulations were tested for their ability to induce both humoral and cellular immunity against MSP1_19_ in mice.

## 2. Results

### 2.1. The SPION-Based DNA Vaccine Formulations: Stability and Uptake by Immune Cells

Throughout our previous studies, we have formulated three different types of magnetic vectors for the purpose of malaria DNA vaccine delivery [14,21,26,27], namely SPOINs/PEI/DNA/HA; SPOINs/PEI/DNA + HA; and SPOINs/PEI + DNA + HA. As summarised in Table 1, among the three magnetic gene vector configurations, the SPOINs/PEI/DNA + HA (second configuration) produced more stability, less particle aggregation, and less toxicity in vitro; therefore, it showed promise as a potential vector for gene delivery in vivo. Further studies on this magnetic gene vector configuration showed that SPIONs/PEI/DNA + HA complexes—containing high molecular weight (MW) of hyaluronic acid (900 kDa) and a high HA:PEI charge ratio (100%)—efficiently transfected DCs in vitro under an external magnetic field. These complexes also induced high levels of expression of activation markers such as major histocompatibility complex (MHC)I, MHCII, and CD86 in vitro (Table 2) [27], making this formulation an ideal candidate to test for use in DNA vaccine delivery. In the present study, we formulated the gene vector configuration with the malaria gene encoding the MSP1_19_ vaccine candidate protein, from *Plasmodium yoelii* (VR1020-PyMSP1_19_ gene complexes) (SPIONs/PEI/DNA + HA (MSP1_19_)), to deliver a malaria DNA vaccine in vivo. We studied the immunogenicity induced by this DNA vaccine formulation using two different routes of administration, intraperitoneal and intramuscular (i.p. and i.m.), with or without the influence of an external magnetic field. Humoral immune responses were assessed by measuring the antigen-specific antibody production by enzyme-linked immunosorbent assay (ELISA), and the upregulation of CD86 on splenic DCs in vivo was evaluated using flow cytometry analysis. Different types of cellular immune responses were quantified by measuring cytokine production elicited from T cells in response to MSP1_19_ by using an enzyme-linked immunospot (ELISpot) assay. The cytokines tested included interferon gamma (IFN-γ), which is characteristic of T helper 1 cells (Th1); interleukin 4 (IL-4), which is produced mainly by Th2 cells; and interleukin 17 (IL-17), which is usually elicited from Th17 cells. 

### 2.2. Antibody Responses Induced by SPIONs/PEI/DNA + HA Complexes

**Figure 1:**
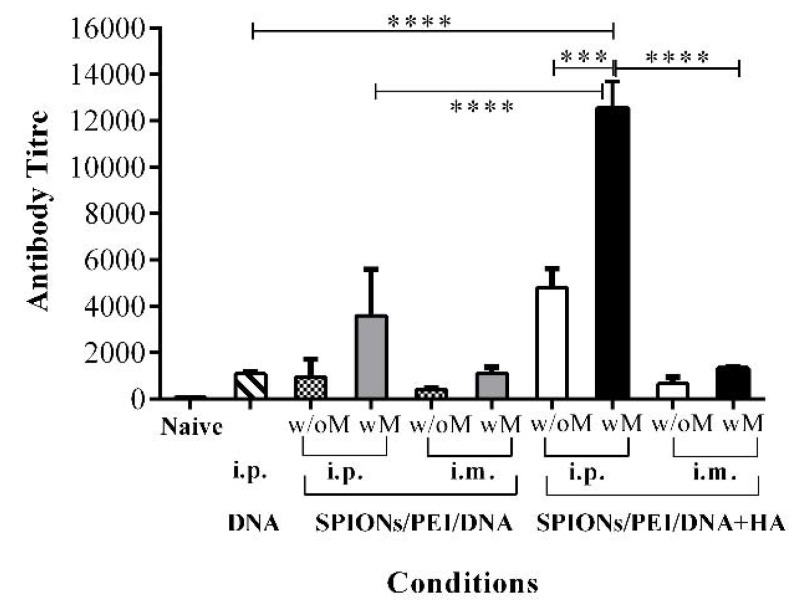
Antibody responses induced by the different magnetic gene complexes compared via different routes of administration. BALB/c mice (*n* = 5/group) were immunised 3 times (3 weeks apart) with SPIONs/PEI/DNA + HA, SPIONs/PEI/DNA, or naked DNA via intraperitoneal (i.p.) and intramuscular (i.m.) administration (naked DNA via i.p. only), with or without the application of an external magnetic field. Two weeks after the final immunisation (day 56), sera were collected and pooled from each group, and measured for total antigen-specific IgG production by ELISA assay, and antibody titres were calculated (see Methods section). Data represented as antibody titre mean ± SD of 2 individual experiments. Statistical significance was designated as *** *p* < 0.001, **** *p* < 0.0001, ((w/M) with magnet, (wo/M) without magnet).

To assess the impact of including HA, the production of PyMSP1_19_ antigen-specific IgG antibody responses were measured in sera from mice immunised with the SPIONs/PEI/DNA + HA complexes in comparison to other configurations, such as DNA alone, or the SPIONs/PEI/DNA configuration. As shown in Figure 1, mice immunised with SPIONs/PEI/DNA + HA complexes via i.p. administration induced significantly higher total IgG antibody responses compared to other DNA complex configurations (e.g., ~4.4-fold higher comparing to DNA alone, and ~5.0-fold compared to the SPIONs/PEI/DNA complex; *p* < 0.001, Figure 1). Such responses were further enhanced with the application of an external magnetic field during vaccine administration (~2.6-fold enhancement with endpoint titre of 12,535, showing an almost ~11.6-fold increase compared to the DNA alone group; *p* < 0.0001, Figure 1). These results suggested that the presence of HA polymer in the gene complexes is essential and responsible for the high antibody responses observed in the SPIONs/PEI/DNA + HA complexes. 

DNA vaccine delivery via i.m. administration induced relatively lower total IgG antibody responses for all formulations tested than i.p. (e.g., antibody titres of 4795 i.p. vs. 665 i.m., *p* < 0.001, Figure 1), and the additional application of an external magnetic field only moderately enhanced the original responses (~1.98-fold, Figure 1) for the SPIONs/PEI/DNA + HA complexes. The DNA alone delivery was only tested by i.p. administration, as it was the best route of administration shown in our previous studies [14].

### 2.3. Antibody Isotypes Induced by the SPIONs/PEI/DNA + HA Complexes

**Figure 2:**
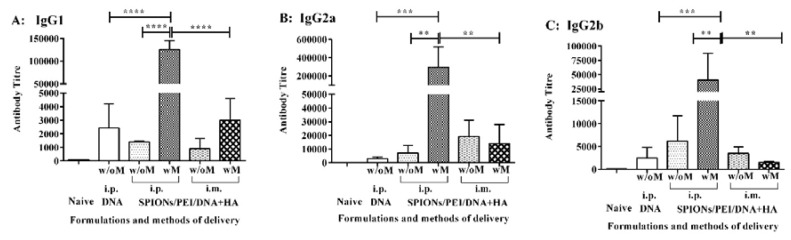
IgG subclasses induced by the SPIONs/PEI/DNA + HA complexes in vivo. BALB/c mice (*n* = 5/group) were immunised 3 times (3 weeks apart) with either SPIONs/PEI/DNA + HA, SPIONs/PEI/DNA, or naked DNA, via i.p. and i.m. administration (naked DNA via i.p. only), with or without the application of an external magnetic field. Two weeks after the final immunisation (day 56), sera from each mouse were collected and measured for antigen-specific IgG1 (**A**), IgG2a (**B**), and IgG2b (**C**) production by ELISA assay. Data represented as antibody titre mean ± SD (*n* = 5 mice). Statistical analysis was performed using one-way analysis of variance (ANOVA) and Tukey’s multiple comparison tests. Statistical significance was designated as ** *p* < 0.01, *** *p* < 0.001, **** *p* < 0.0001, ((w/M) with magnet, (w/o M) without magnet).

The IgG antibody subclass influences their ability to mediate different effector functions such as complement fixation or recognition by Fc receptors on phagocytes [28]. To further evaluate the IgG subclasses induced by the SPIONs/PEI/DNA + HA complexes, sera from the above immunisation studies were further analysed for IgG subclasses. As shown in Figure 2, immunisation with the SPIONs/PEI/DNA + HA complexes induced anti-PyMSP1_19_-specific IgG1, IgG2a, and IgG2b antibodies at different levels. The predominant antibody subclass identified was IgG2a (antibody titre of 295,234; Figure 2B) followed by IgG1 (mean antibody titre of ~125,252; Figure 2A) and IgG2b (mean antibody titre of ~40,644; Figure 2C). The vaccine administration route also influenced the level of antibody production. Although there was a trend for antibody production to increase when the formulation was administrated i.p. rather than i.m., due to substantial variability across individual mice, this trend was not statistically significant. However, the application of an external magnetic field during i.p. injection significantly enhanced antigen-specific antibody levels for all the IgG subclasses tested (i.e., IgG1: ~89.5-fold, IgG2a: ~40.9-fold, and IgG2b: ~6.8-fold, and *p* < 0.0001; *p* < 0.01 respectively, Figure 2).

### 2.4. In Vivo Maturation of Splenic Dendritic Cells Induced by SPIONs/PEI/DNA + HA Complexes Injection

**Figure 3:**
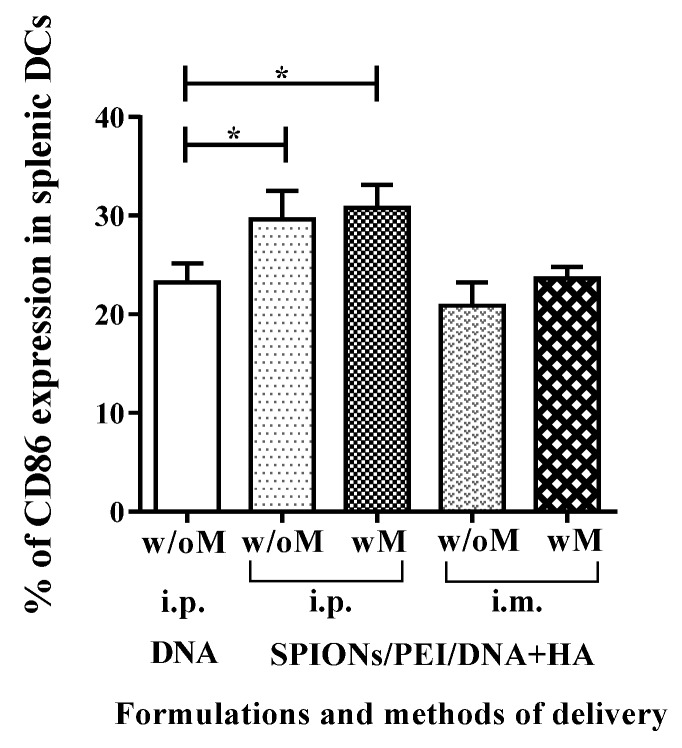
Dendritic cell activation in spleen after injection with SPIONs/PEI/DNA + HA complexes via i.p. or i.m. administration (naked DNA via i.p. only), with or without the application of a magnetic field. Mice (BALB/c) were injected once via i.p. or i.m. route of injection. Forty-eight hours after injection, mice were sacrificed, spleens were harvested, and the levels (%) of CD86 marker in splenic DCs were assessed by flow cytometry. Data are presented as mean percentage of CD86-positive DCs ± SD for each group of treatment (*n* = 3 mice/group). Statistical analysis was performed via *t*-tests, * *p* ≤ 0.05.

The ability of DCs to drive the immune system depends on their functional maturation/activation. Our previous studies had shown SPIONs/PEI/DNA + HA can induce the maturation/activation of DCs in vitro, characterised by upregulation of a number of markers, and particularly the maturation/activation marker CD86 [27]. To confirm that SPIONs/PEI/DNA + HA would similarly interact with, and mature, DCs in vivo, we performed flow cytometry analysis for splenic cells after immunising mice with SPIONs/PEI/DNA + HA complexes via the i.p. and i.m. routes. As shown in Figure 3, immunisation with the SPIONs/PEI/DNA + HA complexes via i.p. route induced phenotypical maturation signals (upregulated CD86 expression) in spleen DCs (defined conventionally as CD11c^+^GR1^−^ splenic cells). No such induction was observed after i.m. injection. This was consistent with the fact that the i.m. injection route limits the migration of APCs to the spleen from the injection site, when compared to other immunisation routes such as subcutaneous, intradermal, or i.p. The application of an external magnetic field had no impact on the magnitude of such induction on either via i.p. or i.m. routes of injections. These results indicated that plasmid DNA loaded onto magnetic nanoparticles can enhance the expression of CD86 in splenic DCs when delivered i.p. 

### 2.5. Cytokine Production Associated with the Cellular-Mediated Immune Responses Induced by the SPIONs/PEI/DNA + HA Complexes 

**Figure 4:**
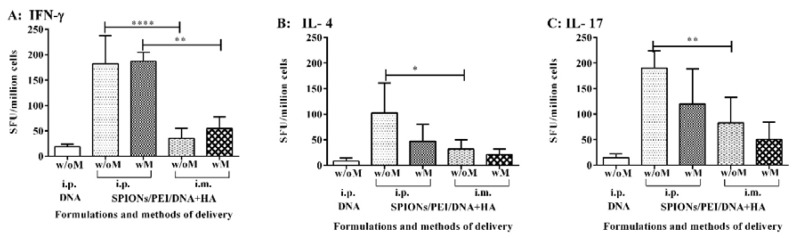
Antigen-specific T cell responses induced by the SPIONs/PEI/DNA + HA complexes in vivo. BALB/c mice (*n* = 5/group) were immunised 3 times (3 weeks apart) with SPIONs/PEI/DNA + HA or naked DNA, via i.p. and i.m. administration, with or without the application of an external magnetic field. Two weeks after the final immunisation (day 56), mice were humanely sacrificed, and splenocytes were harvested and assayed for antigen-specific T cell responses, measured by the induction of IFN-γ, IL-4, and IL-17 cytokine production, upon stimulation with the recall antigen (recombinant EcPyMSP1_19_) in ELISpot assays. All data expressed as mean of spot-forming units (SFU)/million cells ± SD. (**A**) IFN-γ; (**B**) IL-4; (**C**) IL-17 responses. Statistical significance was designated as * *p* ≤ 0.05, ** *p* < 0.01, **** *p* < 0.0001.

Secondary (memory or recall) effector T cell-mediated immune responses to MSP1_19_ induced by the SPIONs/PEI/DNA + HA complexes in vivo were quantified by measuring the production of cytokines induced by MSP1_19_, associated with key subsets of CD4 helper T cells, by ELISpot assay. Cytokines, such as IFN-γ, IL-4, and IL-17, production are representative of Th1, Th2, and Th17 immune responses. BALB/c mice were immunised with the SPIONs/PEI/DNA + HA complexes, either administered i.p. or i.m., with or without further application of an external magnetic field, as above. The antigen-specific T cell responses were measured upon antigen recall with recombinant PyMSP1_19_ protein. As shown in Figure 4A, the antigen-specific IFN-γ responses were predominately induced by the SPIONs/PEI/DNA + HA complexes when administrated i.p., either with or without the addition of a magnetic field. Administration via i.m. and the DNA group alone did not induce any significant IFN-γ responses above the background. Furthermore, contrary to what we had observed previously when measuring the antibody responses, the additional use of the magnetic field did not enhance the observed IFN-γ response. Similarly, levels of the IL-17 (Figure 4B) and lower levels of IL-4 (Figure 4C) were also induced when the SPIONs/PEI/DNA + HA complexes were administrated i.p., whereas the DNA alone group failed to induce IL-4 and IL-17 responses. Moreover, the additional use of a magnetic field did not further enhance the responses observed. Overall, the SPIONs/PEI/DNA + HA complexes were capable of inducing antigen-specific T cell-mediated immune responses, predominantly by Th1 and Th17 cells, and to a lesser extent Th2 cells. Such responses were largely influenced by the formulation delivery methods (route of injection), though the application of an external magnetic field was not required to induce such T cell-mediated responses.

## 3. Discussion

In this study, the malaria gene vector (VR1020-PyMSP1_19_ gene complexes) was incorporated into SPIONs and was shown to be an effective DNA vaccine formulation, capable of inducing combined humoral and cellular immune responses. The nature of the specific nanoparticle DNA can be critical for the induction of such immune responses, specifically, the configuration of the SPIONs. PEI and HA determine the particle size, charge, DNA-binding ability, and stability, as well as DNA transfection efficiency by immune cells (Table 1 and Table 2). Herein, we compared two configurations, SPIONs/PEI/DNA and SPIONs/PEI/DNA + HA, for their ability to induce humoral and cellular immune responses. Our data clearly showed that the SPIONs/PEI/DNA + HA configuration is a potent vaccine for the induction of antibody responses, far superior to the DNA alone (~11.6-fold increases) compared to the SPIONs/PEI/DNA configuration without the addition of HA (~3.5–5-fold increases depending on the application of an external magnetic field). The antibody levels detected here were also greater than other published DNA vaccines using the same malaria construct [29,30]. HA is a high-molecular-weight mucopolysaccharide naturally present in all living organisms [22]. It consists of linear, unbranched polyanions formed by d-glucuronic acid and *N*-acetyl-d-glucosamine repetitive units, which are proven to be low in toxicity, non-antigenic, non-immunogenic, biodegradable and biocompatible. Receptor for hyaluronic acid-mediated motility (RHAMM), as well as TLR4 and TLR2, CD44, TNFIP6, HA receptor for endocytosis (HARE), and lymphatic vessel endothelial hyaluronan receptor 1 (LYVE-1) have been described as the main receptors for HA for different biological functions [23]. In our previous study, we found PEI and HA polymer-coated magnetic gene complexes promoted DC maturation [27]. Maturation of APCs, specifically DCs, is one of the initial events required to elicit potent cellular immune responses. Therefore, the increased potency of the DNA formulation complexes containing HA is potentially due to its ability to promote DC activation during the immune cell priming process. 

The IgG subclass distribution of anti-PyMSP1_19_ antibodies is an important parameter for protection against blood-stage malaria infection [31]. We further studied the antibody IgG subclasses induced by the SPIONs/PEI/DNA + HA complexes. The predominant IgG subclasses induced were found to be IgG2a (antibody titre of 295,234) followed by IgG1 and IgG2b. This result is in agreement with our previous studies, where high-titre IgG2a responses were induced by administration of SPION/PEI/DNA (encoding PyMSP1_19_) complexes [21]. This result is also consistent with the results we obtained here, where higher levels of IFN-γ responses were observed concurrently with the same formulation, since Th1 responses promote IgG2a subclass production over the other subclasses [32,33]. IgG2a cytophilic response type may be the desired type of antibody for vaccine applications against malaria, as this enables effective antibody-dependent cellular cytotoxicity (ADCC), which is an effector response observed in studies that obtained protection against blood-stage malaria [31]. IgG2a antibody production has been linked to protection against *P. yoelii* by several studies [31,34,35]. Other IgG subclasses, IgG1 and IgG2b, are less prominent, nevertheless, high levels of these subclasses were induced by our formulations, with mean antibody titres of ~125,252 and ~40,644, respectively. Both IgG1 and IgG2b antibodies have been associated with low peak parasitemia during malaria infection [36]. The ability to elicit multiple IgG antibody subclasses may therefore be useful for a vaccine aiming to induce protection against blood-stage malaria.

Although antibody responses are important for antimalarial immunity, both cellular and humoral immune responses have frequently been correlated with protection against *Plasmodium* infection. IFN-γ production, as an indicator of Th1 cells, is also associated with blood-stage protection that can eliminate intracellular parasites [37]. Our findings indicated that the SPIONs/PEI/DNA + HA complexes not only induced high levels of antibody responses, but also high levels of Th1 cell responses, and, to a lesser extent, Th2 and Th17 cell responses when injected intraperitoneally, as indicated by the production of IFN-γ, IL4, and IL17 cytokines, respectively. The combination of Th1, Th2, and Th17 responses may also help significantly enhance the host’s ability to combat malaria infections, by engaging multiple effector cell populations. This notion is further supported by the data we showed here. The SPIONs/PEI/DNA + HA complexes was capable of inducing the phenotypical maturation of splenic DCs in vivo, by enhancing the expression of a key T cell-activating molecule, CD86, on the splenic DCs when injected via i.p. route, with or without application of external magnetic field, hence, increasing the T cell-mediated responses. On the contrary, the levels of CD86 expression on the splenic DCs were not changed when the SPIONs/PEI/DNA + HA complexes were injected via the i.m. route, either with or without magnetisation, and were not as able to induce T cell responses. This finding may be explained by the fact that following i.m. injection, DNA mostly transfects myocytes but not DCs, and myocytes can express genes encoded by injected plasmids, but they lack the MHC and costimulatory molecules that are needed as part of T cell activation process [38,39]. This is consistent with our observation of no to low-induction of T cell responses via the i.m. route in the immunogenicity studies herein.

The fact that i.p. administration was superior to i.m. for DNA/nanoparticle complexes is not unexpected, as the i.m. route was chosen for testing since it has been found to be the optimal route for naked DNA injection [38,40], but not for nanoparticle/DNA formulations. When plasmid DNA is made into a particle, the mechanism of transfection becomes evidently different, due to the physiochemical characteristics (e.g., size, shape, and surface charge) of micro- and nanoparticles; these DNA-loaded particles have been shown to be preferentially taken up by APCs over other cells [41]. Therefore, rather than aiming to transfect muscle cells, it targets the transfection of APCs, particularly DCs. Our results support the proof of concept that these nanoparticle formulations can transfect across routes other than i.m., and, specifically via the i.p. route, the nanoparticles/DNA complexes can also enhance the gene transfection of the DCs, upregulating the CD86 expression on DCs and subsequently enhancing the immunogenicity. In addition, the application of a magnet at the injection site for the SPIONs/DNA complexes (also called magnetofection) [42], can also create a “highly concentrated antigen depot” when injected via i.p. route, which is analogous to the well-described “depot” effect, whereby adjuvants retain antigen at a higher localised concentration for subsequent uptake and activation by migratory APCs. “Depot effect” is regarded as one of the mechanisms promoting immunostimulation by adjuvants [43]. The depot effect has traditionally been achieved using lipid-based adjuvants, but in principle, magnetic retention may offer a less intrusive and time-controlled tool to retain antigen in a location conducive to APC exposure. The peritoneal cavity is a major reservoir for macrophages and DCs, the complexes remain in contact with peritoneal cells and lymphoid tissues for long period of time, resulting in more opportunities for cellular uptake of these complexes and generation of long-term DC gene expression and maturation, leading to the induction of potent humoral and cellular immune responses.

The application of external magnet at the injection site after i.m. injection of magnetic particles is expected to cause a different effect than when it is applied after i.p. injection. Due to the physiological barrier of the skeletal–muscle tissue, and dense, thick layers of connective tissues, i.m. injection of magnetic nanoparticles often accumulates in the perimysium space at the injection site, limiting the dispersion of DNA vaccine within muscle [44]. However, upon applying an external magnet at the injection site, magnetic particles/DNA complexes are seen to be more dispersed after i.m. injection with exposure to a magnetic field, comparing it to the nonmagnetic-field exposure [45]. The magnetic field might induce extravasation into surrounding tissue, pulling the particles across the muscle tissues, and resulting in more efficient distribution of DNA within the tissue and rapid sedimentation of the full vector dose on the target cells [45]. This, therefore, increases the gene transfection efficiency in myocytes. However, as discussed previously, myocytes can express genes encoded by injected plasmids, but they lack the MHC and costimulatory molecules that are needed as part of the T cell activation process [38,39]. Our in vivo studies here, comparing the immunogenicity of the SPIONs/PEI/DNA + HA complexes injected via i.p. and i.m. routes, clearly supported the notion of the importance of antigen presentations by APCs rather than by transfected muscle cells in the process of inducing T cell responses. Intraperitoneal injection induced much higher antibody and cell-mediated responses comparing it to the i.m. injection. The limited immune responses (both humoral and cellular) induced by the i.m. injection may due to the limited numbers of APCs (particularly DCs) being transfected across the muscle tissues, therefore affecting the magnitude of the antibody production as well as cytokines production. 

It was also noted that the impact of magnetic field for the antibody production and T cell responses were different. While magnetic field enhanced antibody production greatly, it failed to enhance the T cell responses by any of the routes (i.p. and i.m.) tested here. Although there are no similar studies published in the literature, from other literature evidence, we believe that the lack of effect on the T cell responses after the magnetofection may due to the MSP1_19_ antigen itself. Low response of peptide-specific T cells to MSP1_19_ was observed in response to natural malaria infection [46], and it has been speculated that T cell peptide epitopes may be processed from the protein in low amounts in vivo. T cell responses to reduced recombinant proteins and linear peptides are more prevalent than responses to disulphide-bonded proteins, suggesting that the complex disulphide-bonded structure of native MSP1_19_ may inhibit antigen processing or presentation [47]. Therefore, the failure of further enhancement of T cell responses by magnetofection in our study may partially be due to the complexity of this MSP1_19_ antigen processing and presentation in vivo. Additionally, a recent study using computational approaches to predict epitopes within MSP1_19_ found that B cell epitopes have the lowest energy score compared to the T cell epitopes, indicating a strong binding affinity toward the receptor and, therefore, stronger humoral responses overall [48]. There were six potential B cell epitopes and two potential T cell epitopes predicted as potential candidates for vaccine development against malaria within the MSP1_19_ antigen [48], suggesting a strong humoral-mediated immune response by this antigen. The intrinsic T cell responses may be limited and therefore could not further be enhanced by the magnetofection.

Nevertheless, the present studies showed a novel formulation of a magnetic nanovector and delivery method for the development of DNA vaccines against blood-stage malaria. The present studies also highlight the importance of the DNA vaccine delivery system, and its impact on the induction of desired immune responses, providing further insights of in vivo magnetofection on the impact of the induction of immune responses. The proposed magnetic nanovector formulation holds promise for translating its use in humans. 

## 4. Materials and Methods

### 4.1. Chemical Reagents

Polyethylenimine with an average molecular weight of 25 kDa (PEI branched) and trisodium citrate dihydrate (C_6_H_5_Na_3_O·2H_2_O) were purchased from Sigma-Aldrich (St. Louis, Missouri, MO, USA). Sodium hyaluronate (HA) with an average molecular weight of 900 kDa was purchased from Life Core Biomedical LLC. Fe(III) chloride and Fe(II) chloride were from Ajax Finechem and Ajax Chemicals, respectively. RPMI 1640 medium, 0.05% trypsin-EDTA, penicillin/streptomycin, l-glutamine, and foetal calf serum 2000 were purchased from Gibco- BRL (Gathersburg, MD, USA). 

Mammalian expression vector VR1020 (Vical Inc., San Diego, CA, USA), VR1020-PyMSP1_19_ plasmid was kindly provided by Prof. Ross Coppel’s Group (Department of Microbiology, Monash University). VR1020-PyMSP1_19_ plasmid was amplified in *Escherichia coli* (strain DH5α) and purified using an endotoxin-free Mega-prep plasmid kit (QIAGEN, Hilden, Germany) according to the manufacturer’s instruction. Circular disc Nd–Fe–B permanent magnet (a circular 3 mm × 1 mm disc magnet with a pull strength of 108 g) was purchased from The Aussie Magnet Company Pty Ltd. (Burwood, Victoria, Australia).

### 4.2. Preparation of Plasmids DNA

VR1020-PyMSP1_19_ plasmids were amplified using *Escherichia coli* strain DH5α. A single colony of *E. coli* harbouring plasmid VR1020-PyMSP1_19_ was picked out from a freshly streaked selective plate and inoculated in a 10 mL starter culture medium (LB broth containing 10 g NaCl, 10 g bacto-tryptone, 5 g yeast, and 100 μg·mL^−1^ of kanamycin) for 8 h at 37 °C with vigorous shaking at 200 rpm. The starter culture was then diluted into 1000 mL of LB medium and incubated overnight at 37 °C with vigorous shaking of 200 rpm. The plasmids VR1020-PyMSP1_19_ from *E. coli* cells were purified using the endotoxin-free QIAGEN Mega plasmid purification kit (QIAGEN) according to the manufacturer’s protocol.

### 4.3. Preparation of Magnetic Gene Vectors

Superparamagnetic iron oxide nanoparticles (SPIONs) were synthesised by alkaline coprecipitation of Fe(III) chloride (FeCl_3_·6H_2_O) and Fe(II) chloride (FeCl_2_·7H_2_O) (1:2 molar ratios) in aqueous solution in the presence of trisodium citrate (C_6_H_5_Na_3_O_7_·2H_2_O) as an electrostatic stabiliser, as described in our previous papers [14,21,26,27]. The iron oxide suspension (0.5 mg/mL) was mixed with 10% (*w*/*v*) PEI solution (25 kDa branched polyethylenimine), with PEI/Fe mass ratios (R) = 10, during which they were sonicated for 5 min. To prepare the SPIONs/PEI/DNA complexes, MSP1_19_ plasmid with a total mass of 100 μg/mouse was added to 100 µL of SPIONs/PEI solutions at the molar ratio of PEI nitrogen to DNA phosphate (N/P) of 15. While preparing the SPIONs/PEI/DNA + HA complexes, MSP1_19_ plasmid (with a total mass of 100 μg/mouse) was mixed with equal volumes (e.g., 100 µL) of HA solutions containing HA 900 kDa to form DNA–HA mixture, and then incubated for 10 min at room temperature (RT). At the molar ratio of PEI nitrogen to DNA phosphate (N/P) of 15, DNA–HA matrices were added to 100 µL of SPIONs/PEI solutions. The amount of HA was adjusted according to the 100% charge ratio of –COOH (carboxylic group of hyaluronan) to N (from amine group of PEI). The complexes were then suspended in 5% glucose buffer (water containing 5% glucose) (pH 7.4) for injection.

### 4.4. Immunisation of Mice

Female BALB/c mice aged from 6 to 8 weeks were purchased from the Monash University Animal Services and were kept in a specific pathogen-free (SPF) environment. The immunogenicity study was approved by the Monash University Animal Ethics Committee, Melbourne, Australia. Treatment protocols and care of the animals were in accordance with the Australian Code of Practice for the Care and Use of Animals for Scientific Purposes (Permit number MARP/2011/011). 

6–8-week-old female BALB/c mice were immunised with either SPIONs/PEI/DNA complexes, or SPIONs/PEI/DNA + HA complexes at N/P ratio of 15, or naked DNA in a 5% glucose buffer with PyMSP1_19_, 100 μg/mouse/immunisation. Three injections at 3-week intervals (i.e., at day 0, 21, and 42) were administered. Two injection routes, intramuscular (i.m.) or intraperitoneal (i.p.), as well as the application of an external magnetic field at the injection site for mice receiving the SPIONs formulations, were compared (*n* = 5 mice per experimental condition). To provide an external magnetic field at the injection site, a small neodymium–iron–boron (Nd–Fe–B) permanent magnet (a circular 3 mm × 1 mm disc magnet with a pull strength of 108 g) was tightly attached to the surface of the injection site immediately after injection of SPIONs/PEI/DNA + HA and SPIONs/PEI/DNA complexes for 1 h. Two weeks after the final immunisation (day 56), mice were euthanized by CO_2_ asphyxiation, spleens were removed, and splenocytes were harvested for immunogenicity assays (ELISpot, see below). Sera were also collected before immunisation and at endpoint for detecting antigen-specific antibodies by ELISA.

### 4.5. Antibody Determination by Enzyme-Linked Immunosorbent Assay (ELISA)

To detect PyMSP1_19_-specific antibodies, sera from vaccinated animals were collected before immunisation and at the endpoint, and assayed for antigen-specific antibody production by ELISA. Briefly, 96-well plates (Maxisorp™, NUNC, Roskilde, Denmark) were coated with recombinant EcPyMSP1_19_ (5 μg/mL) in 0.2 M sodium bicarbonate buffer (pH 9.6) and incubated overnight at 4 °C for determination antibody titre and IgG profiles. After washing 5 times with phosphate-buffered saline (PBS)/0.05% Tween-20 and blocking with 5% skim milk in PBS, serial dilutions of mouse sera were added and incubated at 37 °C for 2 h or 4 °C overnight. After washing as above, horseradish-peroxidase (HRP)-conjugated rabbit anti-mouse IgG (Invitrogen, Carlsbad, CA, USA) was added and allowed to incubate at 37 °C for another 1 h. For detection of antigen-specific IgG1, IgG2a, and IgG2b subclasses, a panel of HRP-conjugated rat anti-mouse immunoglobulin subclass IgG1, IgG2a, and IgG2b (BD Biosciences, San Jose, CA, USA) were used at this step. After 1 h incubation, the reaction was developed using TMB (3,3',5,5'-tetramethylbenzidine) substrate (Invitrogen™, Carlsbad, CA, USA) and stopped with 1 M HCl, before reading the absorbance at 450 nm (OD450 nm) using a Multiskan GO microplate reader (Thermo Scientific). The magnitude of the antibody levels was compared by the antibody endpoint titres. The antibody endpoint titres represent the degree to which the serum could be diluted and still contain detectable amounts of antibody, and were calculated as the serum dilution at which the OD450 nm was equal to the mean OD of the serum of naïve mice + 3 standard deviations (SD).

### 4.6. Flow Cytometry

To analyse the phenotype and activation status of the splenic DCs, spleens were isolated 48 h from mice after injection with SPIONs/PEI/DNA + HA or naked DNA, via i.p. and i.m. administration, with or without the application of an external magnetic field, and they were processed into single-cell suspension. The cells were incubated with ACK lysis buffer for 1 min at room temperature to the lyse red blood cells. The remaining leukocytes were counted and stained with the following antibodies: anti-CD11c V450 (HL3), anti-Gr1 PerCP Cy5.5 (RB6-8C5), and biotinylated anti-CD86 (GL1), whereas dead cells were excluded with live/dead fixable dead cell stain kit (Aqua LIVE/DEAD, Invitrogen). Biotin-labelled monoclonal antibody was developed with streptavidin APC and analysed on the BD LSRII. All antibodies were purchased from BD Pharmingen unless otherwise stated. DCs were defined as CD11c^+^ Gr1^−^ cells as described [49].

### 4.7. ELISpot Assay

Antigen-specific immune responses to MSP1_19_ were evaluated by IFN-γ, IL-17, and IL-4 ELISpot assays. Briefly, 96-well filtration plates (Multiscreen TM sterile plate from Millipore, Billerica, MA, USA) were coated with 100 µL/well of either anti-mouse IFN-γ (mAb AN18, 5 µg/mL MABTech, Stockholm, Sweden), anti-mouse IL-17 (mAb IL-17-I, 5 µg/mL, MABTech), or rat anti-mouse IL-4 (mAb, 5 µg/mL, BD Pharmingen, San Diego, CA, USA). Following overnight incubation at 4 °C, the wells were washed with PBS and blocked with complete media (CM, RPMI 1640 completed medium supplemented with 10% heat-inactivated foetal bovine serum (FBS), 20 mM HEPES, 4 mM glutamine, 100 units/mL penicillin, 100 µg/mL streptomycin, and 0.4 mM 2-mercaptoethanol). Splenocytes from immunised mice (1 × 10^7^ cells/mL, 50 µL) were added to triplicate wells of each type of cytokine-precoated plates (IFN-γ, IL-17, and IL-4, as above) and co-cultured with 50 µL of recall antigens (25 µg/mL, recombinant EcPyMSP1_19_ (*Escherichia coli* PyMSP1_19_)). All plates were incubated at 37 °C in an incubator filled with 5% CO_2_ for a minimum of 16 h for IFN-γ plates, 24 h for IL-4 plates, and 36 h for IL-17 plates. Concanavalin A (Con-A) (1 µg/mL final, Amersham Biosciences, Uppsala, Sweden) was used as a positive control and background wells were added with CM. Following incubation, the plates were then washed 5–6 times in PBS and incubated with 100 µL biotinylated detection antibodies (anti-mouse IFN-γ biotinylated mAb R4-6A2 (MABTech), or anti-mouse IL-17 biotin (mAb IL-17-II, MABTech), or rat anti-mouse IL-4 biotin (mAb BVD6-24G2, BD)) (all at 1 µg/mL final), at room temperature for 1–2 h. After washing as above, streptavidin–alkaline phosphatase (ALP) (to detect IFN-γ and IL-17) or ExtrAvidin–ALP (for detection of IL-4) were added (final at 1 µg/mL) and incubated for a further 1–2 h at room temperature. Plates were then washed again, with a final wash using Reverse Osmosis (RO) water to remove residual PBS. The spots were developed using a colourimetric Bio-Rad AP conjugate substrate kit (Bio-Rad, Philadelphia, PA, USA) following the manufacturers’ instructions. Spot counting was performed using an AID ELISpot Reader System (AID GmbH, Strasberg, Germany). The amounts of the specific cytokine induction in response to the recall antigen were compared for their spot-forming units (SFU). 

### 4.8. Statistical Analysis

Statistical analysis was done by one-way or two-way ANOVA and the Tukey post-test using Graph Pad Prism v6.04 software (GraphPad Software, Inc., La Jolla, CA, USA). Differences were considered statistically significant at *p* < 0.05. Group sizes are indicated in the figure legends. All values are expressed as mean ± SD.

## 5. Conclusions

Overall, our findings suggested that SPIONs/PEI/DNA + HA complexes induced a mixed Th1/Th2/Th17 response with a predominantly Th1 response. The high levels of IgG2a, IgG1, and IgG2b antibodies induced by this formulation under a magnetic field offer a potential way forward to explore further improvement in DNA vaccine delivery. Altogether, our study suggests that the use of magnetic nanoparticles for malaria gene delivery can induce a broad spectrum immune response and substantially enhance the potency of traditional DNA vaccines.

## Figures and Tables

**Table 1:** Summary of properties of different magnetic gene vector configurations.

Test Conditions	Buffer Conditions	Formulation Configurations		
		1st Configuration (SPIONs/PEI/DNA/HA)	2nd Configuration (SPIONs/PEI/DNA + HA)	3rd Configuration (SPIONs/PEI + DNA + HA)
Size (nm) (with the increase % of charge ratio HA:PEI)	water	100–300	100–300	400–200
	NaCl	1000–500	>1000	500–2500
	RPMI	600–200	500–100	400–1600
	RPMI + 10% FCS	150–40	150–40	180–70
Charges (with the increase % of charge ratio HA:PEI, reduced the positive charges)	water	~32 mV to ~−25 mV	~32 mV to ~−25 mV	~29 mV
	NaCl	~14 mV to ~−10 mV	~9 mV to ~−10 mV	~17 mV to ~2 mV
	RPMI	~8 mV to ~−12 mV	~8 mV to ~−15 mV	~12 mV to ~−5 mV
	RPMI + 10% FCS	increase the positive charge (~−18 mV to ~−10 mV)	increase the positive charge (~−18 mV to ~−10 mV)	increase the positive charge (~−16 mV to ~−8 mV)
DNA retardation (with the increase % of charge ratio HA:PEI)	water	>25% DNA released	partial DNA disassembly, but no free DNA	no DNA release or disassembly
	NaCl	No release of DNA	No release of DNA	No release of DNA
	RPMI	No release of DNA	No release of DNA	No release of DNA
	RPMI + 10% FCS	partial DNA disassembly	partial DNA disassembly	no DNA release or disassembly
DNA binding	5% of charge ratio HA:PEI	96% ± 3%	94% ± 1%	99% ± 0.5%
	100% of charge ratio HA:PEI	95% ± 2%	93% ± 3%	99% ± 1%
Stability against extracellular environment		+	+++	++
Stability against nuclease		+	+++	++

Abbreviations—SPIONs: superparamagnetic iron oxide nanoparticles; PEI: polyethylenimine; HA: hyaluronic acid; FCS: foetal calf serum

**Table 2:** The impact of molecular weight (MW) of HA and the HA:PEI ratio on particle uptake and regulation of dendritic cells (DCs) (for particle configuration: SPIONs/PEI/DNA + HA).

	HA Molecular Weight (MW)	HA:PEI Ratio
Efficiency of particle uptake	increase with higher MW	increase with higher ratio
Impact of external magnetic field on uptake efficiency	increase with lower MW formulation	increase with lower ratio formulation
CD86 expression	increase with higher MW	increase with higher ratio
Major histocompatibility complex I (MHC I) expression	MHC I upregulation only observed in 100% HA under magnetic field	no upregulation at lower ratio either with or without magnetic field
Major histocompatibility complex II (MHC II) expression	MHC II upregulation only observed in 100% high MW HA under magnetic field	no upregulation at lower ratio either with or without magnetic field

## Abbreviations

The following abbreviations are used in this manuscript.

APC	antigen presenting cells
DC	dendritic cells
ELISA	enzyme-linked immunosorbent assay
ELISpot	enzyme-linked immunospot assay
HA	hyaluronic acid
i.p.	intraperitoneal
i.m.	intramuscular
MSP	merozoite surface protein
PEI	polyethylenimine
SPIONs	superparamagnetic iron oxide nanoparticles
